# Detection of differentially culturable tubercle bacteria in sputum using mycobacterial culture filtrates

**DOI:** 10.1038/s41598-021-86054-z

**Published:** 2021-03-22

**Authors:** Bhavna G. Gordhan, Julian S. Peters, Amanda McIvor, Edith E. Machowski, Christopher Ealand, Ziyaad Waja, Neil Martinson, Bavesh D. Kana

**Affiliations:** 1 Department of Science and Technology/National Research Foundation Centre of Excellence for Biomedical TB Research, School of Pathology, Faculty of Health Sciences, University of the Witwatersrand and the National Health Laboratory Service, P. O. Box 1038, Johannesburg, 2000 South Africa; 2 Perinatal HIV Research Unit, Faculty of Health Sciences, University of the Witwatersrand, Johannesburg, South Africa; 3 Center for Tuberculosis Research, Johns Hopkins University, Baltimore, MD USA

**Keywords:** Tuberculosis, Translational research

## Abstract

Rapid detection of tuberculosis (TB) infection is paramount to curb further transmission. The gold standard for this remains mycobacterial culture, however emerging evidence confirms the presence of differentially culturable tubercle bacteria (DCTB) in clinical specimens. These bacteria do not grow under standard culture conditions and require the presence of culture filtrate (CF), from axenic cultures of *Mycobacterium tuberculosis* (*Mtb*), to emerge. It has been hypothesized that molecules such as resuscitation promoting factors (Rpfs), fatty acids and cyclic-AMP (cAMP) present in CF are responsible for the growth stimulatory activity. Herein, we tested the ability of CF from the non-pathogenic bacterium *Mycobacterium smegmatis* (*Msm*) to stimulate the growth of DCTB, as this organism provides a more tractable source of CF. We also interrogated the role of *Mtb* Rpfs in stimulation of DCTB by creating recombinant strains of *Msm* that express *Mtb rpf* genes in various combinations. CF derived from this panel of strains was tested on sputum from individuals with drug susceptible TB prior to treatment. CF from wild type *Msm* did not enable detection of DCTB in a manner akin to *Mtb* CF preparations and whilst the addition of RpfAB^*Mtb*^ and RpfABCDE^*Mtb*^ to an *Msm* mutant devoid of its native *rpfs* did improve detection of DCTB compared to the no CF control, it was not statistically different to the empty vector control. To further investigate the role of Rpfs, we compared the growth stimulatory activity of CF from *Mtb*, with and without Rpfs and found these to be equivalent. Next, we tested chemically diverse fatty acids and cAMP for growth stimulation and whilst some selective stimulatory effect was observed, this was not significantly higher than the media control and not comparable to CF. Together, these data indicate that the growth stimulatory effect observed with *Mtb* CF is most likely the result of a combination of factors. Future work aimed at identifying the nature of these growth stimulatory molecules may facilitate improvement of culture-based diagnostics for TB.

## Introduction

The global tuberculosis (TB) epidemic remains a leading cause of death with an estimated 10 million people afflicted with the disease and 1.5 million deaths annually^1^. Moreover, a large proportion of people harbour the infectious agent, *Mycobacterium tuberculosis* (*Mtb*), in lung lesions without any symptoms, providing a massive reservoir of the pathogen that can reactivate to cause active disease and drive further transmission. TB is curable however, the 6 month protracted treatment regimen is difficult to implement and challenges related to patient adherence result in the development of drug resistant forms of the pathogen that cannot be eradicated using the current standard first line drugs^2^. Although several clinical trials have investigated novel treatment shortening therapies, published trials have yet to show broad benefit, possibly due to limited understanding of mycobacterial growth states during active and asymptomatic or subclinical disease.

Several studies recently have provided evidence that sputum from TB patients with active disease is characterised by a large fraction of non-replicating bacteria^3–6^. A commonly used diagnostic method for detection of *Mtb* in patient sputum is enumerating mycobacterial colony forming units (CFU) of culture dilution suspensions on solid agar media. However, this methodology is limited to detecting only replicating mycobacterial populations and is unable to account for the non-replicating population, which has important implications for TB diagnostics. This is illustrated by prior work that demonstrated the presence of differentially culturable tubercle bacteria (DCTB) in sputum that do not grow on solid media and are detected only in liquid media supplemented with culture filtrate (CF)^3,6^. This demonstrates that TB infection presents with a much higher level of bacterial phenotypic complexity than previously thought and a deeper understanding of the clinical relevance of DCTB populations is vital for the advancement of improved TB diagnosis and development of novel drug regimens^4^.

The growth stimulatory activity of CF has been ascribed to the presence of resuscitation promoting factors (Rpfs), a group of secreted enzymes in *Mtb*^6,7^ however, use of this approach is limited by the availability of freshly prepared *Mtb* CF in diagnostic laboratories. To investigate alternative approaches for detection of DCTB, we explored the use of CF from *Mycobacterium smegmatis* (*Msm*), a non-pathogenic close relative of *Mtb,* with the hypothesis that *Msm* CF would provide a more safer and practical approach in routine laboratories. Bioinformatic analysis identified three *rpf* genes with a duplicate *rpfE* homolog (*rpfA*, *rpfB*, *rpfE1* and *rpfE2*) in *Msm* compared to the five homologous (*rpfA*-*rpfE*) in *Mtb*^8^ (Figure S1). In prior work, we generated a mutant of *Msm* devoid of all Rpfs^9^ and herein, we exploited this strain to generate complemented mutant strains that carry *Mtb* Rpfs in various combinations. These recombinant strains were used to generate CF for detection of DCTB in sputum. We found that *Mtb* CF was superior at detecting DCTB when compared to CF from *Msm* and whilst the addition of *Mtb* Rpfs to *Msm* did improve recovery of DCTB, the bacterial yield was not comparable to that observed with *Mtb* CF. It has also been demonstrated that fatty acids and cyclic-AMP (cAMP) are able to stimulate the growth of DCTB in an in vitro model of mycobacterial dormancy^10^. Hence, we also tested various fatty acids and cAMP for the ability to resuscitate DCTB and found that no single agent was able to stimulate bacterial growth in a manner comparable to *Mtb* CF.

## Results

### Construction of *Msm* mutant strains expressing *Mtb rpf* genes

**Figure 1:**
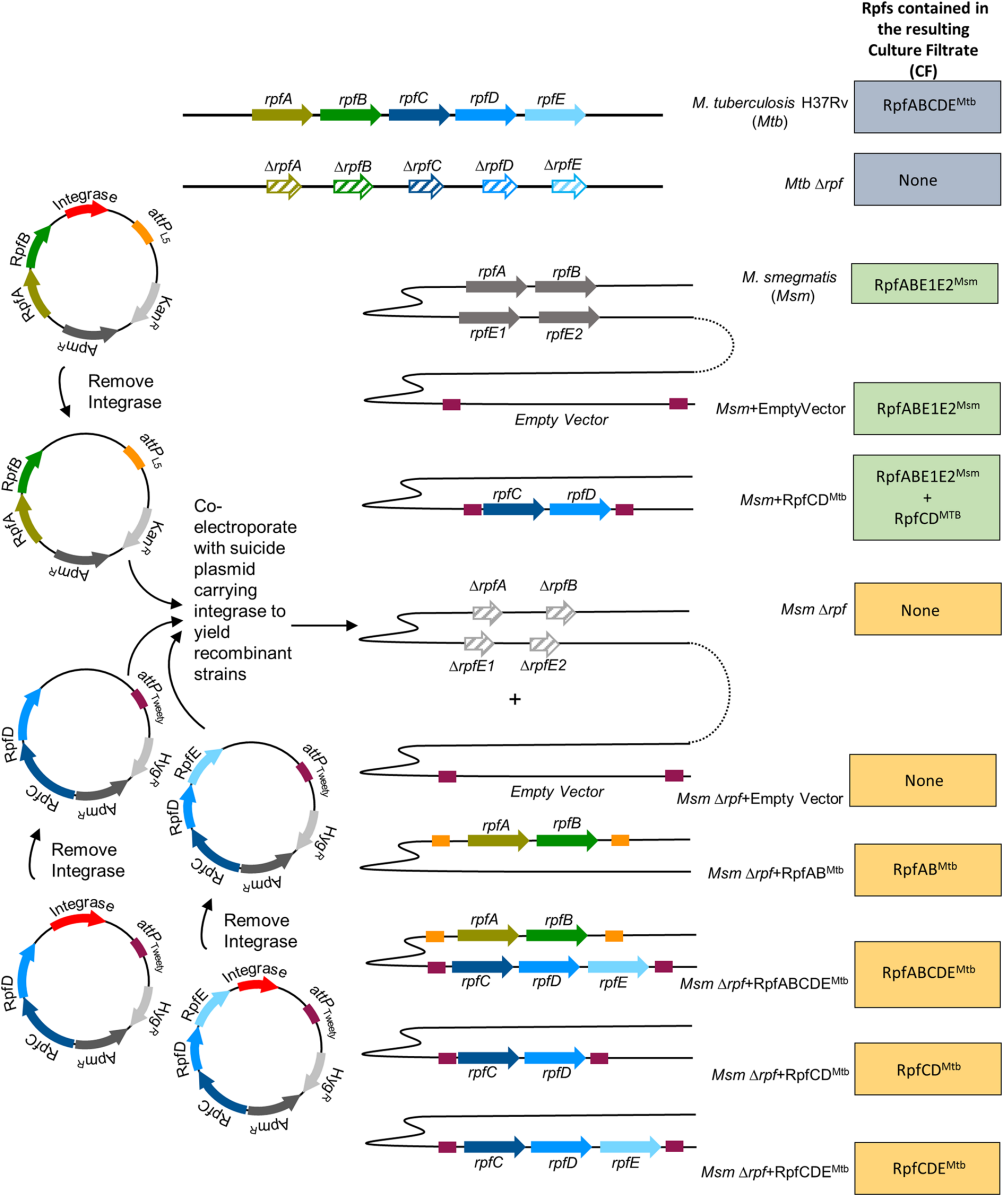
Strategy for construction of recombinant *Msm* strains expressing *Mtb* Rpfs in various combinations. The *Mtb* H37Rv wild type and Rpf defective mutant (*Mtb* ∆*rpf*) were used as a source of *Mtb* culture filtrates (CFs). Wild type *Msm* was used as a source of *Msm* Rpfs and this strain was transformed with a vector carrying RpfCD^*Mtb*^ to mimic the Rpf complement from *Mtb*. The *Msm* mutant defective for Rpfs (*Msm* ∆*rpf*) was used as a control and within this mutant background, vectors carrying various combinations of *Mtb* Rpfs were inserted to create strains that express *Mtb* Rpfs in *Msm* CF as a source of growth stimulatory molecules in MPN DCTB assays. The panel on the right shows the *rpf* gene complement present in the various *Mtb* and *Msm* strains. The vector diagram on the left depicts the strategy to construct vectors with different combinations of *Mtb* Rpfs that are stably carried in the resulting recombinant strains. The presence of the integrase on mycobacterial vectors that integrate phages at attachments sites, facilitates the first integration event but then can also lead to excision of the vector in the absence of antibiotic selection. For DCTB assays, CF that is free of antibiotic was required as antibiotics will inhibit the growth of sputum-derived bacteria. To facilitate this, the integrase was first removed from vectors expressing *Mtb* Rpfs and was provided *in trans* on another suicide vector during transformation. With this approach, the integrase is present for the first integration event and is then lost as it does not carry a mycobacterial origin of replication.

To assess the role of *Mtb* Rpfs in stimulation of DCTB, we generated strains of *Msm* that expressed *Mtb rpf* genes. The inclusion of antibiotics during the culture of these strains to ensure that *rpf*-expressing plasmids are retained was problematic as the antibiotic would be carried into the resulting CF, leading to inhibition of bacterial growth in sputum cultures. To address this, we used integrating plasmids as these carry an integrase to facilitate plasmid integration into the bacterial chromosome at phage attachment sites^11,12^. However, these integrases can also spontaneously excise vectors in the absence of antibiotic selection. To overcome this, we generated versions of conditionally integrative plasmids that lacked the integrase gene and then co-electroporated these with the suicide plasmid that provided the integrase *in trans* (Fig. 1). With this approach, the integrase is present during the initial integration event and is then lost during subsequent cell division cycles. We used a mutant of *Msm*, deleted for all four of the *Msm rpf* genes to build recombinant strains expressing the different *Mtb rpf* genes. The resulting strains were then used to generate CF from strains expressing *rpf* genes from *Mtb* for use in the MPN assay for the detection of DCTB. Wild type *Msm*, carrying the *Msm* Rpfs was also used as a control. The strains generated and predicted complement of Rpf proteins from the resulting CF are shown in Fig. 1.

**Figure 2:**
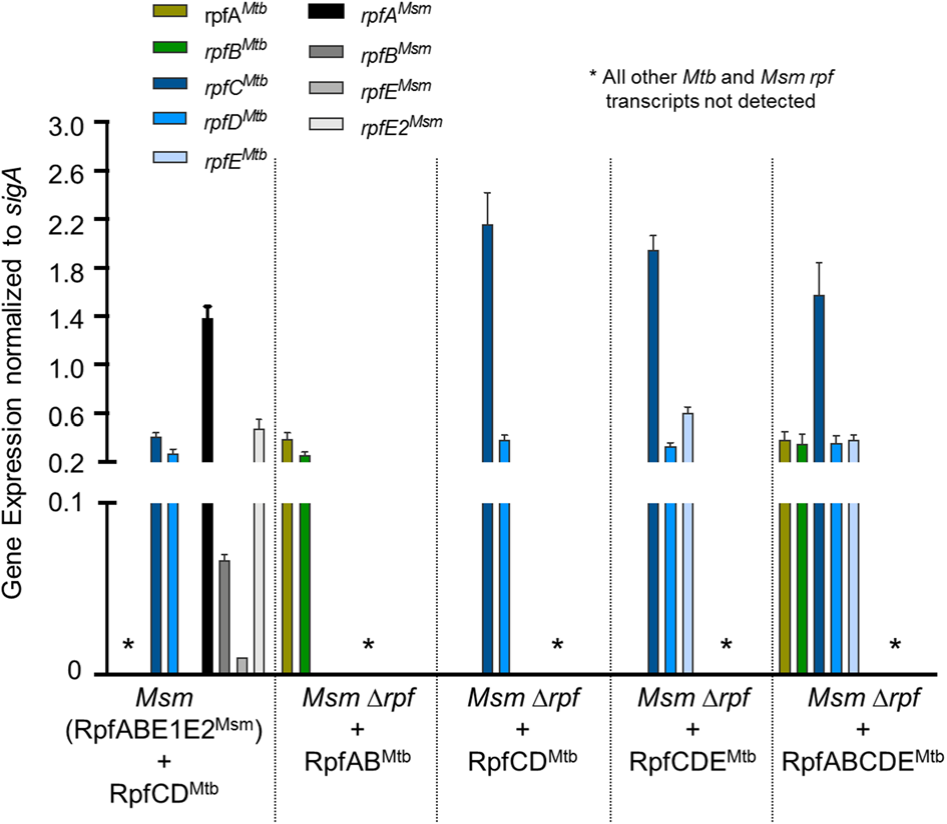
Assessment of *Mtb* and *Msm rpf* gene expression in recombinant strains. Transcriptional analysis was carried out on recombinant strains of *Msm* and the cognate parent strains. In all strains shown, transcriptional analysis was carried out for all five *Mtb* and four *Msm rpf* genes. Where these genes were deleted, no transcripts were detected. Transcript levels were assessed in each strain when axenic cultures reached mid-exponential growth (OD_600nm_ ~ 0.6). Gene expression was normalized against *Msm sigA* transcript levels. Data are representative of three independent biological repeats. Graph was generated using Graphpad Prism software.

To confirm that the *rpf*-like genes from *Mtb* are expressed in *Msm* from the modified integrative plasmids, we conducted transcriptional analysis using quantitative real-time PCR. Recombinant *Msm* strains expressed all *Mtb rpf* genes provided on integrative plasmids. With the exception of *rpfC*^*Mtb*^, all *Mtb rpf* genes were expressed at equivalent levels, Fig. 2. As we had used native *Mtb* promoters, we expected some difference in *rpf* gene expression in recombinant *Msm* strains. As the *Mtb* genes were expressed in *Msm*, we also assessed expression of *Msm rpf*-like genes for comparative purposes. The *rpfA*^*Msm*^ gene was expressed at the highest level relative to the other *Msm rpf*-like genes and the *rpfE2*^*Msm*^ gene was expressed at levels similar to the *rpfABDE*^*Mtb*^ genes.

We anticipated that both recombinant *Mtb* and *Msm* Rpfs would be secreted as analysis of the domain structure of these proteins confirmed the presence of a SecA-dependent signal sequences in all of them, except *Mtb* RpfC (Figure S1). We further confirmed the presence of these signal peptides using Signal P analysis, which identified clear Sec-dependent signal peptides in all proteins, except RpfC (Figure S2). In addition, both SecA1 and SecA2 are conserved in *Msm* and *Mtb* (Figure S3), which when combined with the demonstrated secretion of Rpfs in prior work^13^, suggests that recombinant Rpfs are most likely secreted from the cell and these proteins, or the products of their catalytic activity, are present in the CF used in this study.

### Quantification of DCTB in sputum specimens using different mycobacterial CF preparations

**Figure 3:**
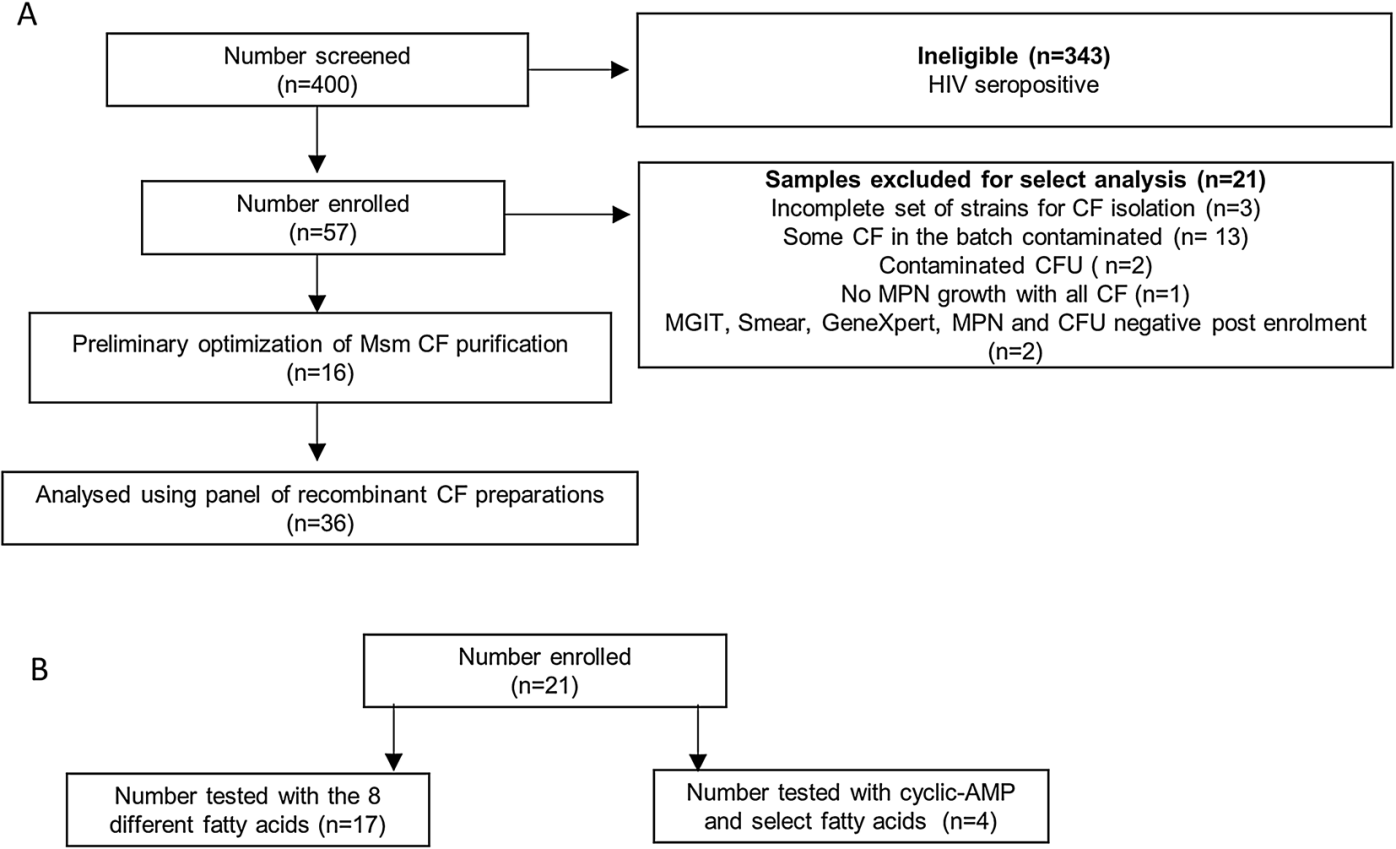
Participant disposition flow chart for individuals recruited to this study. (**A**) Sputum specimens obtained from these individuals were decontaminated and the resulting bacteria grown in limiting dilution assays with various CF combinations. (**B**) Sputum specimens obtained from these individuals were decontaminated and the ability of the various fatty acids and cyclic-AMP to resuscitate the DCTB was assessed by MPN and CFU assays.

To assess the capacity of CF from the various mycobacterial strains generated to detect DCTB, we established a cross-sectional observation cohort of HIV-negative individuals with drug susceptible TB. The participant disposition flow chart for our cohort is given in Fig. 3A. We screened 400 individuals and enrolled 57 individuals. A large number of screen failures were due to co-incident HIV infection and these were excluded from our study due to the low bacterial burden that is expected to prevail in sputum. A summary of participant demographics and laboratory data is included in Table 1. All included participants were sputum smear positive and the majority had medium or high bacterial loads by GeneXpert, with a median time to culture positivity in the MGIT system of 5.5 days.

**Table 1:** Demographics and laboratory diagnostic data for participant specimens tested with various CFs and FAs.

Variables	Measurement with various CF (n = 36)	Measurement with various FA (n = 21)
**Demographics**		
Age, yr, Median (IQR)	33 (25.25–46.75)	35 (30.5–41.5)
**Conventional TB diagnosis, n (%)**		
Smear grade negative	0	1 (4.8)
Smear grade positive	36 (100)	20 (95.2)
Scanty/+	10 (27.8)	6 (28.6)
++	7 (19.4)	5 (23.8)
+++	19 (52.8)	9 (42.9)
**GeneXpert result**		
High, n (%)	17 (47.2)	9 (42.9)
Medium, n (%)	13 (36.1)	10 (47.6)
Low, n (%)	6 (16.7)	1 (4.8)
Very low, n (%)	0	1 (4.8)
None, n (%)	0	0
Median (IQR) GeneXpert cycle threshold	17.35 (15.1 – 21.1)	17 (14.7–20)
**MGIT Time to Positivity, d, median (IQR)**	5.5 (3.0 -7.8)	6 (4.5–8)
**HIV Status**		
**Negative (%)**	36 (100)	8 (38.1)
**Positive (%)**	0	13 (61,9)

*yr* years, *d* days, *IQR* interquartile range, *MGIT* mycobacterial growth indicator tube.

**Figure 4:**
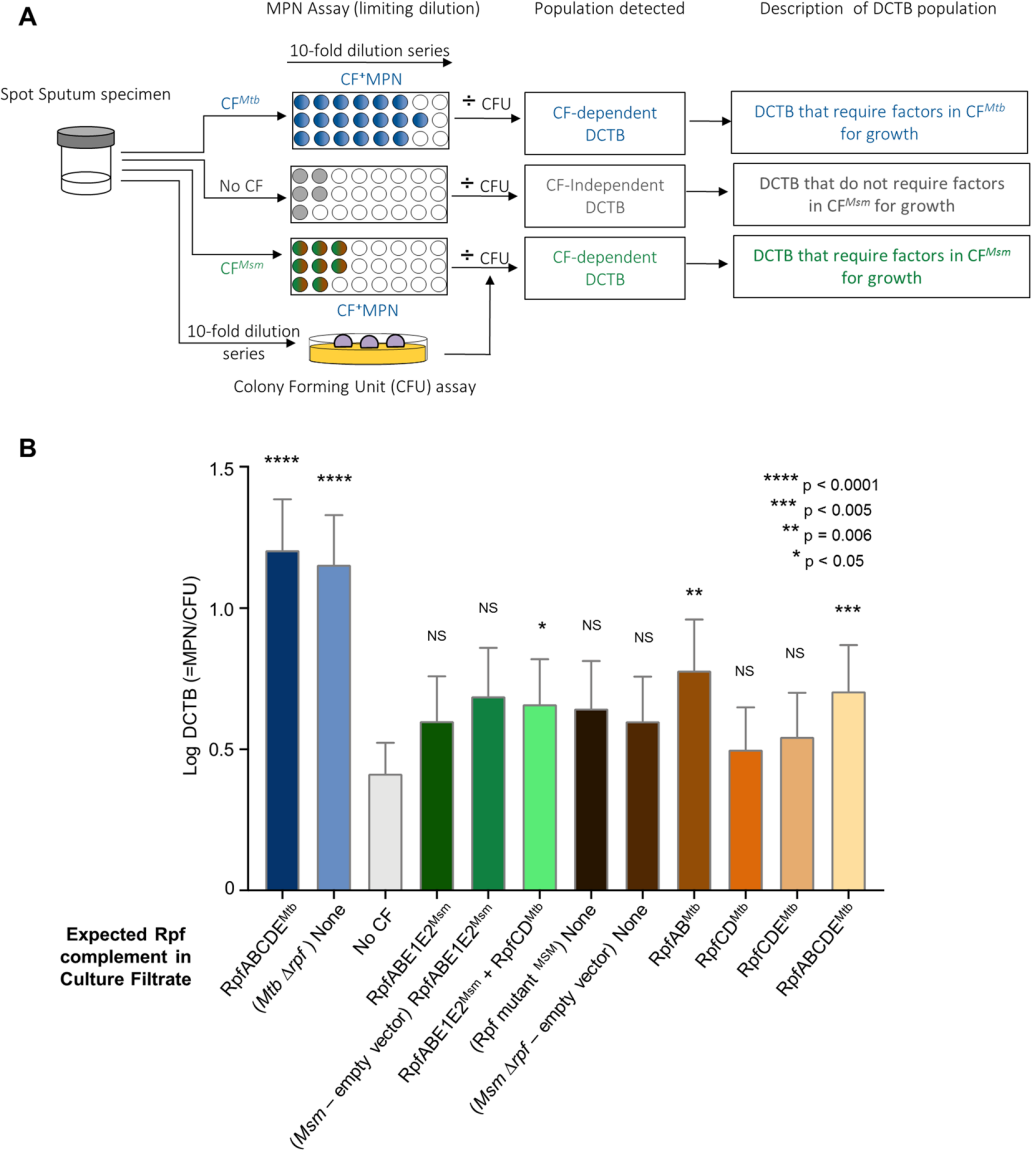
Bacterial yield in DCTB assays using CF from *Msm* strains expressing *Mtb* Rpfs in various combinations. **(A**) Assessment of DCTB recovery from sputum cultures using CF supplementation. DCTB were detected using the MPN limiting dilution assay. For comparison, CF containing and deficient in *Mtb* and *Msm* Rpf was used to assess the role of Rpfs in growth stimulation. To control for the effect of CF in growth stimulation, fresh Middlebrook media was used. CF from various recombinant *Msm* strains was used to assess if expression of *Mtb* Rpfs in *Msm* yields CF that can be used to stimulate DCTB in sputum specimens in a manner that is comparable to *Mtb* CF. To obtain the DCTB count, MPN values (with or without CF) were divided by CFU counts, which depict conventionally culturable bacteria. (**B**) Histogram depicting mean DCTB counts from assays using CF from *Mtb* and *Msm.* The mean DCTB counts were compared to that obtained from no CF assays using a Wilcoxon test to determine the growth stimulatory effect of the various CFs. Error bars depict the standard error of the mean. P-values depict the comparison of any given CF to the no CF or empty vector control. Graph was generated using Graphpad Prism software, Version 6.

Sixteen specimens were used for methods optimization as we had contamination of the CF with bacteria, requiring iterative revision of the filtering protocol. Two specimens yielded contamination on CFU plates, 1 specimen gave no *Mtb* on MPN assays and 2 specimens were negative for MGIT, Smear, GeneXpert, MPN and CFU after enrolment (Fig. 3A). After these exclusions, 36 specimens were tested and analysed for DCTB using the approach outlined in Fig. 4A. As reported previously^3^, we were able to demonstrate that inclusion of *Mtb* CF resulted in detection of higher number of specimens with DCTB (24/36), together with a higher DCTB quantum when compared to using media with no CF (13/36), Fig. 4B. We next sought to determine if addition of Rpfs from *Mtb* to *Msm* would yield a CF with comparable growth stimulatory properties as that obtained from *Mtb*. The genome of *Msm* encodes four *rpf*-like genes, *rpfA*, *rpfB*, *rpfE1* and *rpfE2*^8^. As there were no counterparts for *rpfC*^*Mtb*^ and *rpfD*^*Mtb*^ in *Msm*, we first inserted these two genes into wild type *Msm* to create a CF that  would contain all five mycobacterial Rpfs, RpfABCDE, by combining *Msm* and *Mtb* homologues (albeit with a duplicate copy of *rpfE1* [*rpfE2*]). When compared to the no CF control, inclusion of CF from wild type *Msm* in MPN assays did not yield a comparable increase in DCTB quantum and appeared to be indistinguishable from assays with no CF. The addition of RpfCD^*Mtb*^ to *Msm* yielded a significant increase in the mean quantum of DCTB, Fig. 4B, but this was not associated with detection of DCTB in more specimens when compared to the no CF control (both yielding 13/36 DCTB positive specimens).

Following this, we next asked if CF derived from *Msm* containing only *Mtb* Rpfs was able to stimulate growth of DCTB, as *Msm* would be a more tractable source of Rpfs in the diagnostic setting. For this, we used our previously reported mutant of *Msm* deleted for all four *Msm rpf*-like genes^9^, *Msm* Δ*rpf*, and inserted *Mtb rpf* genes into this genetic background in different combinations. We found that only the addition of RpfAB^Mtb^ and RpfABCDE^Mtb^ to *Msm* Δ*rpf* yielded a marginal but statistically significant increase in the quantum of DCTB recovered, together with a statistically non-significant increase in the number of positive specimens detected (15/36 for both compared to 13/36 for the no CF control). However, whilst the mean DCTB values for CF containing RpfAB^Mtb^ and RpfABCDE^Mtb^ were higher than the empty vector control (mean DCTB yield of 0.8 log and 0.7 log for RpfAB^Mtb^ and RpfABCDE^Mtb^ compared to 0.6 log for the empty vector control), the difference was not statistically significant. Addition of RpfCD^Mtb^ and RpfDCE^Mtb^ to the *Msm* Δ*rpf* mutant did not yield CF preparations that were able to stimulate more DCTB when compared to the no CF control (Fig. 4B).

Data from our heterologous complementation experiments suggested that addition of *Mtb* Rpfs to *Msm* does not yield growth stimulatory effects comparable to that seen with the *Mtb* CF, suggesting that other factors could also contribute to the growth stimulatory effect. To further investigate this, we compared DCTB yields using *Mtb* CF derived from wild type *Mtb* or a mutant defective for the five *rp*f genes. There was no statistical difference in the quantum of DCTB detected with CF derived from these two strains.

### Assessment of the ability of fatty acids and cyclic-AMP (cAMP) to resuscitate DCTB

We next set out to evaluate whether other molecules present in the CF are able to contribute to the growth stimulatory effect seen with sputum specimens and tested the ability of free fatty acids and cAMP to stimulate DCTB. For this component of the study, we obtained sputum (n = 21) from a second collection of patients with drug susceptible TB of whom, 62% were HIV-coinfected (Table 1). The participant disposition flow chart and demographics, together with laboratory data, are shown in Fig. 3B and Table 1 respectively. Twenty specimens were smear positive, the majority had high or medium bacterial load as assessed by GeneXpert, with a median time to positivity on MGIT culture of 6 days. Of these, 4 specimens were used to interrogate the effect of cAMP and select fatty acids, whilst the remainder were used for further analysis of a broader range of fatty acids.

**Figure 5:**
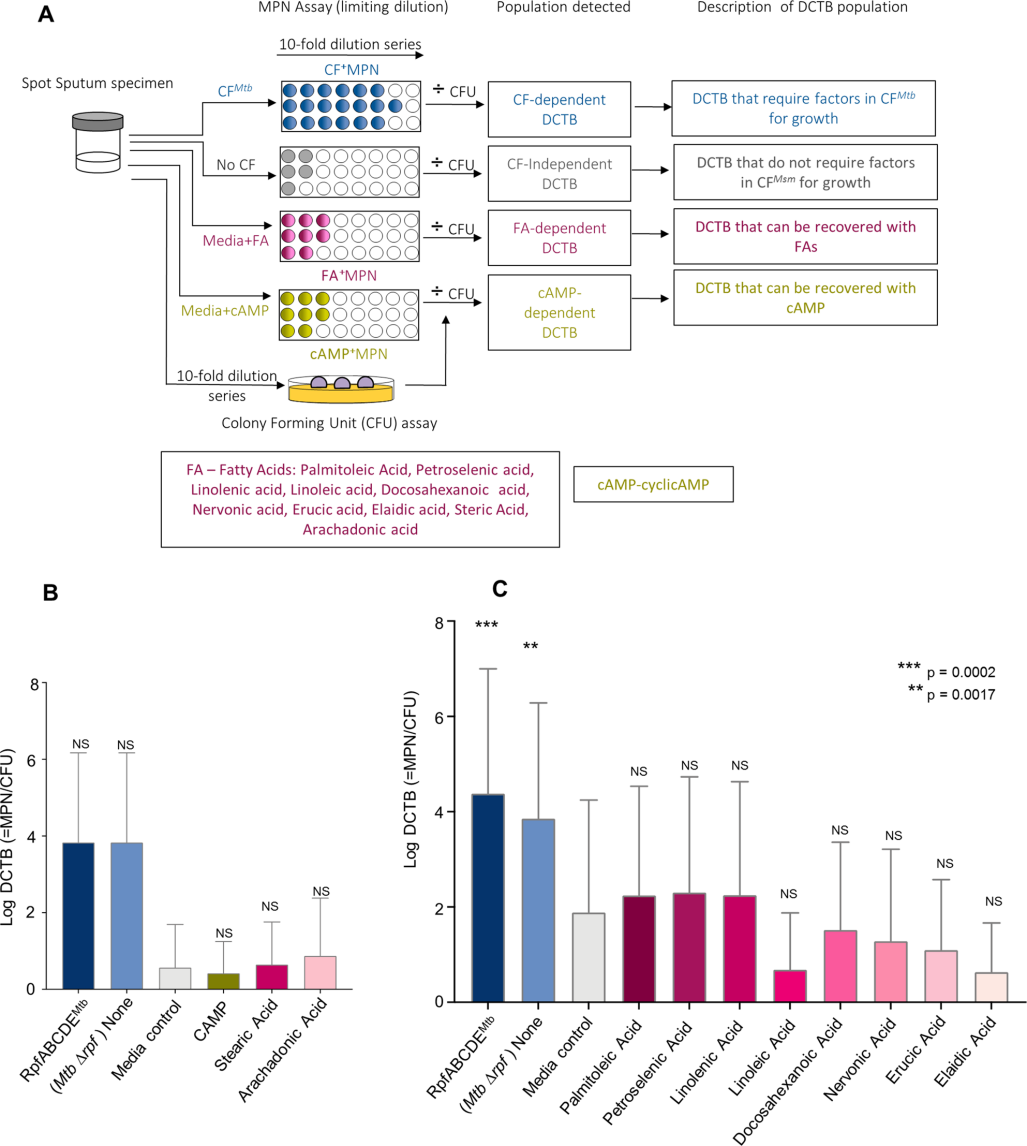
Bacterial yield in DCTB assays using CF containing Fatty acids and cyclic-AMP. (**A**) Assessment of DCTB recovery from sputum cultures using CF, fatty acid and cyclic-AMP (CAMP) supplementation. DCTB were detected using the MPN limiting dilution assay. For comparison, CF containing and deficient in *Mtb* Rpfs was used to compare the growth stimulatory effects of fatty acids and cAMP. To control for the effect of CF in growth stimulation, fresh Middlebrook media was used, which standardly contains oleic acid. To obtain the DCTB count, MPN values (with or without CF) were divided by CFU counts, which depict conventionally culturable bacteria. (**B**) Histogram depicting mean DCTB counts from MPN assays supplemented with standard media containing cAMP or select fatty acids. Statistical comparisons were conducted between CFs and the no CF or cAMP/fatty acid assays. There was an increase in bacterial recovery from MPN assays containing CF, which was not significant, most likely due to the small samples size (n = 4), no significant increase in DCTB recovery was noted in the media control and cAMP or fatty acid assays. Error bars depict the standard error of the mean. (**C**) Histogram depicting mean DCTB counts from assays using CF from *Mtb* and standard media supplemented with fatty acids*.* The mean DCTB counts were compared to that obtained from the media controls assay using a Wilcoxon test to determine the growth stimulatory affect of fatty acids. P-values depict the comparison of the CFs to the no CF or fatty acid assays. Graphs were generated using Graphpad Prism software, Version 6.

In prior work, cAMP appeared to facilitate recovery of DCTB from an in vitro model of differential culturabilty, together with fatty acids such as arachidonic acid, whereas addition of stearic acid did not yield the same effect^10^. Hence, we first tested these agents (as outlined in Fig. 5A) in a small selection of sputum specimens and found that neither cAMP nor arachidonic acid facilitated recovery of DCTB in a manner comparable to *Mtb* CF (Fig. 5B). We also noted that stearic acid did not enhance recovery of DCTB (Fig. 5B). Following this, we opted to test a broader range of fatty acids, with the hypothesis that sputum resident organisms may respond to different stimuli when compared to differentially culturable bacteria generated in vitro. We chose a variety of chemically distinct molecules and first optimised the concentrations using the previously reported in vitro model of *M. smegmatis* differential culturability^14^. The concentration that yielded the best bacterial recovery (detailed in Table S1) were then applied to sputum DCTB assays (Fig. 5A). Certain fatty acids such as palmitoleic, petroselenic and linolenic acid yielded a marginal, statistically non-significant increase in bacterial recovery when compared to media alone (Fig. 5C). However, no fatty acid yielded growth stimulation in a manner comparable to CF from *Mtb*. We noted that in this collection of sputum specimens, the yield of DCTB with CF from *Mtb* was higher when compared to yields shown in Figure 4B. As these specimens were collected from two distinct cohorts of individuals, at different times, these differences may relate to sputum collection procedures or individual characteristics of the participants.

## Discussion

A growing number of studies point to the presence of a differentially culturable population of tubercle bacteria in sputum which require CF to grow in liquid or on solid media^3,5,15,16^, this growth stimulatory effect of CF has been ascribed to the presence of Rpfs^6,15^. A single Rpf was identified in *Micrococcus luteus* as an essential secreted protein able to stimulate the growth of dormant bacterial cultures and represented a group of bacterial growth modulators^17^. Subsequent studies have highlighted a role for these proteins in exit from dormancy in vitro and during virulence and reactivation in the murine model of TB infection^18–21^. It has been shown that these proteins exert their effects through breakdown of the peptidoglycan component of the cell wall, leading to the release of growth stimulating muropeptides suggesting that the growth stimulatory effect can be applied to bacterial cultures in a paracrine manner, through supplementation of growth media with the Rpf proteins themselves^22,23^. Consistent with this, it has been demonstrated that mycobacterial Rpfs are secreted into the culture media hence, making the CF amenable for the identification of DCTB^13^.

In a previous study we demonstrated the utility of CF derived from *Mtb* to detect DCTB in individuals from whom the sputum specimen yielded negative cultures using standard techniques^3^. This suggested that CF-supplementation in the diagnostic setting could improve TB detection in difficult to diagnose cases with paucibacillary TB however, use of *Mtb* CF is limited by the requirement to perform these processes in containment laboratories and need for expert skills to culture the pathogen with subsequent separation of the CF from the bacteria. In this context, *Msm* provide a tractable avenue to generate CF but it remained unclear if this would yield a similar growth stimulatory effect. As the genome of *Msm* lacks homologues for *rpfC* and *rpfD*, we first added the *rpfC*^*Mtb*^ and *rpfD*^*Mtb*^ genes into the genome of wild type *Msm*, to create a CF that was comparable to that derived from *Mtb*. Thereafter, we tested various combinations of *Mtb rpf*-genes in the *Msm* Δ*rpf* mutant for growth stimulatory capabilities. None of the CF preparations from *Msm* yielded growth stimulatory effects that were comparable to CF from *Mtb*.

The addition of CF containing RpfAB^*Mtb*^ and RpfABCDE^*Mtb*^ yielded a small but significant increase in recovery of DCTB and given that addition of CF with RpfCDE^*Mtb*^ did not yield comparable effects, the increased growth stimulatory effect by the former CF may be due to RpfAB^*Mtb*^. These results should be interpreted with caution as in cases where addition of select *Mtb* Rpfs appeared to significantly increase the yield of DCTB compared to the no CF control, these differences were not significantly different when compared to CF from the no vector control strain. In our prior work, we demonstrated that the combinatorial effect of RpfA and RpfB from *Msm* played an important role in biofilm formation^9^, suggesting that these two proteins may have an essential function in bacterial communication.

In addition to the Rpf proteins, cAMP and fatty acids have also been reported to stimulate the growth of dormant *Msm*^10^. These factors in CF, together with other as yet unidentified molecules may work in concert to allow for paracrine stimulation of DCTB in sputum specimens. We tested the effect of cAMP and a variety of fatty acids and whilst these appear to differentially facilitate recovery of differentially culturable *Msm* in vitro, they had no benefit in recovery of DCTB in sputum. When combined with the observations on recombinant CFs (with and without *Mtb* Rpfs), our data suggests that the growth stimulatory effect observed in sputum specimens cultured with *Mtb* CF is most likely the result of a combination of factors. Further work to identify the interplay between these factors and how they enhance bacterial growth may yield useful new supplements for diagnostic media and would facilitate the development of faster culture-based diagnostics for TB.

## Materials and methods

All methods were performed in accordance with the relevant guidelines and regulations for growth of *Mtb* and handling of human specimens. All procedures were conducted in a BioSafety Level III laboratory, registered with the South African Department of Agriculture Forestry and Fisheries (Registration Number: 39.2/NHLS-20/010). All procedures were approved by the Institutional BioSafety Committee of the University of the Witwatersrand (approval number: 20200502Lab).

### Bacterial strains and culture conditions

Bacterial strains and plasmids used in this study are listed in Table 2. *E. coli* and *M. smegmatis* strains were grown as previously described^9^.

**Table 2:** Plasmids and strains used in this study.

Plasmids^a^	Description, markers and/or genotype
pHINT^11^	L5 mycobacteriophage-based integrating shuttle phasmid; *bla*, *hyg*, *attP*_L5_ and integrase gene *int*; AP^R^, Hyg^R^
pMV-RPFAB^9^	L5 mycobacteriophage-based integrating shuttle phasmid based on pMV306 containing *rpfA* and *rpfB*; *hyg*, *attP*_L5_-*int,* Hyg^R^
pH-RPFCD^21^	L5 mycobacteriophage-based integrating shuttle phasmid based on pHINT containing *rpfC* and *rpfD; bla, hyg*, *attP*_L5_-*int,* AP^R^, Hyg^R^
pH-RPFCDE^21^	L5 mycobacteriophage-based integrating shuttle phasmid based on pHINT containing *rpfC*, *rpfD* and *rpfE*, *bla*, *hyg*, *attP*_L5_-*int,* AP^R^, Hyg^R^
pTTP1B^12^	Tweety mycobacteriophage-based integrating shuttle phasmid; *bla*, *aph*, *attP*_Tweety_ and integrase gene *int;* AP^R^, Km^R^
pTT-RPFAB	pTTP1B derivative. *rpfA* and *rpfB* from pMV-RPFAB cloned as an *Mlu*I-*Bst*BI fragment; *bla*, *aph*, *attP*_Tweety_-*int,* AP^R^, Km^R^
pTT-RPFABΔi	pTT-RPFAB derivative. Internal *Nco*I deletion in the *int* gene. *rpfA*, *rpfB*, *bla*, *aph*, *attP*_Tweety,_ AP^R^, Km^R^
pH-RPFCDΔi	pH-RPFCD derivative. Internal *Hin*dIII to *Bam*HI deletion of the *rpfE* and *int* genes. *rpfC*, *rpfD*, *bla, hyg, attP*_L5_, AP^R^, Hyg^R^
pHRPFCDEΔi	pH-RPFCDE derivative. Internal *Hin*dIII to *Bsr*GI deletion in the *int* gene. *rpfC*; *rpfD*; *rpfE; bla*; *hyg*; *attP*_L5;_ AP^R^, Hyg^R^
pHINT- attP-hyg	pHINT based integrating derivative. *Hin*dIII to *Nde*I excision of the *int*_L5_ gene; *bla*, *hyg*, *attP*_L5_, AP^R^, Hyg^R^
pHINT-int-bla	pHINT-based suicide plasmid. *Eco*RI to *Nde*I excision of *attP*_L5_ and *hyg bla*; *int*_L5_*,* AP^R^
pTTP1B-int-bla	pTTP1B-based suicide plasmid. *Hin*dIII to *Acc*I (partial) excision of *attP*_Tweety_ and *aph*. *bla*, *int*_Tweety_*,* AP^R^
**Strains**	
*E. coli* DH5a	Strain used for routine cloning; *supE44* Δ*lacU169 (*ϕ*80 lacZ*Δ*M15*) *hsdR17 recA1 endA1 gyrA96 thi-1 relA1*
*M. smegmatis* mc^2^155 (*Msm*)^25^	*ept-1* (efficient plasmid transformation) mutant of mc^2^6
*Msm* Δ*rpf* ^9^	*M. smegmatis* quadruple *rpf* deletion mutant carrying internal in-frame deletions in *rpfA*, *rpfB*, *rpfE1* and *rpfE2*
*M. tuberculosis* H37Rv (*Mtb*)	Virulent reference laboratory strain (ATCC 25,618)
*Mtb* Δ*rpf* ^21^	*M. tuberculosis* quintuple deletion mutant carrying internal in-frame deletions in *rpfA*, *rpfC*, *rpfB*, *rpfE* and *rpfD*
*Msm* + RpfCD^Mtb^	*Msm* carrying *rpfC* and *rpfD* in pH-RPFCDΔi and integrated at the *attP*_L5_ site, Hyg^R^
*Msm* + Empty Vector	*Msm* carrying the vector control pHINT-attP-hyg with no *rpf* genes and integrated at the *attP*_L5_ site, Hyg^R^
*Msm* ∆*rpf* + RpfAB^Mtb^	*Msm* ∆*rpf* carrying *rpfA* and *rpfB* in pTT-RPFABΔi and integrated at the *attP*_Tweety_ site, Km^R^
*Msm* ∆*rpf* + RpfCD^Mtb^	*Msm* ∆*rpf* carrying *rpfC* and *rpfD* in pH-RPFCDΔi and integrated at the *attP*_L5_ site, Hyg^R^
*Msm* ∆*rpf* + RpfCDE^Mtb^	*Msm* ∆*rpf* carrying *rpfC*, *rpfD* and *rpfE* in pH-RPFCDEΔi and integrated at the *attP*_L5_ site, Hyg^R^
*Msm* ∆*rpf* + RpfABCDE^Mtb^	*Msm* ∆*rpf* carrying *rpfA* and *rpfB* in pTT-RPFABΔi integrated at the *attP*_Tweety_ site and *rpfC*, *rpfD* and *rpfE* in pH-RPFCDEΔi integrated at the *attP*_L5_ site, Km^R^, Hyg^R^
*Msm* ∆*rpf* + Empty Vector	*Msm* ∆*rpf* carrying the vector control pHINT-attP-hyg with no *rpf* genes and integrated at the *attP*_*L5*_ site, Hyg^R^

*Ap*^*R*^ ampicillin-resistant, *Hyg*^*R*^ hygromycin-resistant, *Km*^*R*^ kanamycin-resistant.

^a^Where relevant, the reference for the plasmid/strain is given. In other cases, the plasmid/strain originated from laboratory stocks or this work.

### Cloning of shuttle plasmids for integration into *M. smegmatis*

To prevent spontaneous excision of the integrated vector in the absence of antibiotic selection, previously generated integrating vectors carrying the *rpf* genes^21^ were manipulated to remove the integrase gene. Plasmid pMV-RPFAB was digested with *Mlu*I and *Bst*BI and the 6313 bp blunt ended fragment was cloned into pTTP1B at the *Acc*65I site to generate pTT-RPFAB. The resulting plasmid was subsequently partially digested with *Nco*I, and the 10,937 bp fragment was circularised to yield plasmid pTT-RPFABΔi (Table 2). Plasmids pH-RPFCD and pH-RPFCDE^21^ were digested with *Hin*dIII/*Bam*HI and *Hin*dIII/*Bsr*GI respectively and the resulting 9496 bp and 10,907 bp fragments were blunt ended and circularised to yield plasmids pH-RPFCDΔi and pH-RPFCDEΔi (Table 2). The empty vector control, pHINT-attP-hyg, containing no *rpf* genes was generated by digesting pHINT with *Hin*dIII/*Nde*I and the resulting 4781 bp fragment, was blunted and circularised (Table 2). The integrase protein was provided in *trans*, by generating suicide plasmids from pHINT and pTTP1B which lack the phage attachment sites *attP*_L5_ and *attP*_Tweety_, respectively (Table 2). The pHINT was digested with *Eco*RI/ *Nde*I, and pTTP1B was digested with *Hin*dIII and partially with *Acc*I to generate the 4204 bp fragment and the 4533 bp fragment respectively. These fragments were blunt ended and circularised to yield plasmid pHINT-int-bla and pTTP1B-int-bla (Table 2). These suicide vectors bearing the integrase gene of either pHINT or pTTP1B were co-electroporated with the conditionally integrating plasmids carrying the *rpf* genes in various combinations into *Msm* and the *Msm* Δ*rpf* mutant to generate recombinant strains of *Msm* carrying various combinations of *Mtb rpf* genes, detailed in Table 2. Hygromycin or kanamycin resistant transformants were picked and screened for expression of the corresponding *Msm* and *Mtb rpf* genes by RT-PCR using the primers listed in Table S1.

### Gene expression analysis by real time, qRT-PCR

To monitor the expression of the *rpf* genes, all recombinant strains were grown to early exponential phase (OD_600nm_ 0.6). Gene expression analysis was conducted as previously described^9^ using primers indicated in Table S2.

### Recruitment of participants to obtain sputum specimens

Ethics approval for participant recruitment was obtained from the Human Research Ethics Committee of the University of the Witwatersrand, South Africa, with clearance number M110833, subsequently revised to M200164 (SNT study). Individuals 18 years and older attending the Perinatal HIV Research Unit (PHRU), Soweto, South Africa were approached for participation into the study. Fifty seven HIV sero-negative patients with drug sensitive TB, as determined from either an auramine stained smear or by GeneXpert obtained from the public sector (National Health Laboratory Service, Johannesburg, South Africa) were eligible for enrolment in the study. Patients willing to participate were approached and written informed consent was obtained using Informed Consent Forms reviewed and approved by the Human Research Ethics Committee of the University of the Witwatersrand. After written informed consent was granted, a spot sputum was collected for analysis by the most probable number (MPN) assay (otherwise referred to as limiting dilution assays) as described previously^3^. The MPN assay is a limiting dilution series based on a Poisson distribution for the quantification of bacterial growth in liquid media, described in further detail in the Supplementary Information. Sputum samples were serially diluted in a 48 well microtitre plate in media with culture filtrate (1:1 ratio) purified from wild type *Msm, Mtb* or a variety of mutant/recombinant strains. Where relevant, fatty acids or CAMP were added to media in the MPN assay.

### Optimization of fatty acid concentrations to use in sputum DCTB assays

The concentrations of fatty acids for use in sputum were optimised using an in vitro model of dormancy that generates differentially culturable *Msm* as previously described^14^. *Msm* was pre-cultured in 20 ml of nutrient rich broth for 16 h at 37 °C in an orbital shaker (250 rpm). Pre-cultures were sub-cultured into modified Hartman’s–de Bont medium. Trace elements solution was prepared as previously described^24^. The OD_600nm_ of the pre-culture was adjusted to 0.9 and 20 ml was added to 130 ml of the Hartmans de Bont media and incubated at 37 °C for 14 days without shaking. The unsaturated fatty acids kit (Sigma Catalogue number UN10-1KT) was used for the resuscitation of differentially culturable *Msm*. Each fatty acid was initially resuspended in 1 ml of dimethyl sulfoxide to create a working stock. The starved *Msm* was added to Middlebrook media (supplemented with ADC—no oleic acid was used) and the OD_600nm_ adjusted to 0.1. Thereafter, 9 ml aliquots of these cells was used to test the different concentrations of fatty acids. Fatty acids were tested at the following concentrations: 0.125 µM, 0.25 µM, 0.5 µM, 1 µM, 2.5 µM, 5 µM, 7.5 µM, 10 µM, 12.5 µM, 15 µM, 17.5 µM, 20 µM, 22.5 µM, 25 µM, 27.5 µM, 30 µM and 32.5 µM. The OD_600nm_ of each culture was assessed after 72 h and the bacterial recovery was measure as the fold increased in OD_600nm_ compared to the inoculum. For cAMP (3 mM), steric acid (4 µM), arachinodic acid (1.6 µM) and Linoleic acid (1.7 µM) the concentrations used were derived from previous similar work^10^.

## Data analysis

MPN values from assays containing distinct CF preparations were compared in a pairwise fashion using a student’s t test. All statistical analysis was done using GraphPad Prism software, version 6.

## Supplementary information

Supplementary information.
