# Supplementary material for: Detection of differentially culturable tubercle bacteria in sputum using mycobacterial culture filtrates
Source: Sci Rep. 2021 Mar 22;11:6493. doi: 10.1038/s41598-021-86054-z (PMC7985135; doi:10.1038/s41598-021-86054-z)
Supplement: Supplementary file 1 — Supplementary information. [file 41598_2021_86054_MOESM1_ESM.docx]

**Detection of differentially culturable tubercle bacteria in sputum using mycobacterial culture filtrates**

Bhavna G. Gordhan^1#^, Julian S. Peters^1#^, Amanda McIvor^1#^, Edith E Machowski^1#^, Christopher Ealand^1^, Ziyaad Waja^2^, Neil Martinson^1,2,3^ and Bavesh D. Kana^1*^

**Supplementary Information**

^1^Department of Science and Technology/National Research Foundation Centre of Excellence for Biomedical TB Research, School of Pathology, Faculty of Health Sciences, University of the Witwatersrand and the National Health Laboratory Service, Johannesburg, South Africa; ^2^Perinatal HIV Research Unit, Faculty of Health Sciences, University of the Witwatersrand, Johannesburg, South Africa; and ^3^Center for Tuberculosis Research, Johns Hopkins University, Baltimore, Maryland

^#^ These authors contributed equally to this work.

Keywords: tuberculosis, differentially culturable tubercle bacteria (DCTB), culture filtrate (CF), resuscitation promoting factors (Rpfs), limiting dilution assays

*For correspondence: Mailing Address: DST/NRF Centre of Excellence for Biomedical TB Research, National Health Laboratory Service, P. O. Box 1038, Johannesburg 2000, South Africa. Phone: Tel: + (27) 11 489 9030; Fax: + (27) 11 4899397; E-mail: [bavesh.kana@wits.ac.za](mailto:bavesh.kana@wits.ac.za) (BDK)

**Supplementary Methods**

**Recruitment criteria**

Two distinct collections of sputum specimens were used in this study. For the analysis of recombinant CFs, a cohort of patients was recruited with inclusion criteria indicating participants had to be ≥18 years and able to produce at least 3 ml of sputum. Only HIV-uninfected individuals were recruited to this component of the study. The participants had to be willing to provide an address or mobile phone number where they could be contacted if the smear and /or mycobacterial culture was positive for TB and they were not already on TB treatment. Institutionalized persons (e.g. prisoners, long term hospitalization [>1 month] or living in an institution) and patients on multidrug TB treatment were excluded. All patient names were anonymised and given a unique study identity. Spot sputum samples were collected and shipped on that day to the laboratory for decontamination and downstream processing.

For analysis of cAMP and fatty acids, a second collection of sputum specimens was used. In this case, recruitment criteria and all recruiting procedures were the same as for the first collection of specimens with the exception that HIV-infected individuals were included and contributed the majority of specimens (62%) used.

**Decontamination of sputum samples**

A 1:1 volume of 2.9% sodium citrate and 4% sodium hydroxide was added to the sputum sample and incubated at room temperature for 20 min followed by centrifugation at 3900xg for 10 min. The cell pellet was washed with 45 ml of 0.01 M phosphate buffered saline (PBS), pH 7.4 and the pellet resuspended in 2 ml of Middlebrook 7H9 media supplemented with 10% OADC, 0.2% glycerol and 0.05% Tween80 (7H9). Half this volume was used for the Most Probable Number (MPN) analysis and CFU assessment and the remainder was frozen at -80°C.

**Bacterial culturing and culture filtrate (CF) preparation procedures**

Cultures of *M. tuberculosis* (*Mtb*) strains were grown by inoculating 1 ml freezer stocks (OD_600nm_ 0.5-0.8) into 8 ml of 7H9 and grown for 2.5 days to an OD_600nm_ of ~0.5. The pre-culture was added to 42 ml of 7H9 media and allowed to grow for 2-3 days to an OD_600nm_ of 0.6-0.8. A similar procedure for *M. smegmatis* (*Msm*) strains was followed except that 1 ml of the respective freezer stock (OD_600nm_ 0.5-0.8) was added to 25 ml of 7H9 supplemented with 0.085% NaCl, 0.2% glucose, 0.2% glycerol and 0.05% Tween80, grown for 3 days to an OD_600nm_ of 0.6-0.7 and used for culture filtrate (CF) preparation. CF was obtained by centrifugation of the cultures for 10 min at 3900x g and filtration of the supernatants through two 0.22 μM filters attached in tandem using a 50 ml syringe. CF from both the *Mtb* and *Msm* strains was prepared in real time for each batch of sputum samples. The filtered CF was diluted with 50% 7H9 media and supplemented with 8% (w/v) PANTA (polymyxin, amphotericin B, naladixic acid, trimethoprim and azlocillin, Becton Dickinson, South Africa) to manage contamination.

To confirm sterility of the CF, 1 ml aliquots of each was incubated at 37 °C for 3 months with an additional aliquot spread on 7H11 media and the plates incubated at 37 °C for 8 weeks. Aliquots of the CF from both *Msm* and *Mtb* were also screened by PCR for select *rpf* genes using *Mtb rpf* gene specific primers^1^ and *Msm* *rpf* gene specific primers^2^ to confirm strain genotypes and to ensure no cross contamination of strains occurred during CF preparation.

**The MPN assay**

The MPN assay was performed as previously described ^3,4^. Briefly, 450 μl of each of the PANTA supplemented CFs or media containing the various fatty acids or cAMP at the appropriate concentration were dispended across 3 columns (triplicate) in all wells in a 48 well microtitre plate (Figure 4A). Media supplemented with PANTA was used as the control in triplicate in the remaining three columns. The 1 ml decontaminated sputum sample was transferred to a 50 ml Falcon tube containing 0.5 ml of sterile 2 mm glass beads and vortexed for about 30 seconds to break up bacterial clumps/cords. Fifty microlitres of the sputum sample was added to all the wells in the first row and 10-fold dilutions were carried out across the plate until the last row from which 50 μl of sample was discarded (Figure 4A). The plates were sealed with biohazard tape and incubated at 37 °C for six weeks, followed by visual scoring of growth using an inverted mirror. The total number of bacteria in the MPN assay was estimated using software available at <http://www.wiwiss.fu.berlin.de/fachbereich/vwl/iso/ehemalige/wilrich/index.html>.

**Determination of colony forming units (CFUs)**

The CFU’s were determined by spreading appropriate 10 fold dilutions of the decontaminated sputum onto Middlebrook 7H11 media supplemented with 0.5% glycerol and 10% OADC (Figure 4A). The plates were incubated at 37 °C for four weeks before scoring colonies.

**Bioinformatic analysis of the *Msm* and *Mtb* Rpfs**

Protein sequences for all nine genes (5 *Mtb* Rpf and 4 *Msm* Rpf homologues) were obtained from Mycobrowser in FASTA format (<https://mycobrowser.epfl.ch/>). These were used as input to identify domains at InterPro 83.0 (<https://www.ebi.ac.uk/interpro/>). Further analysis was done to confirm the output from InterPro annotation: SignalP V5.0 for secretion signals (<http://www.cbs.dtu.dk/services/SignalP/>), PFAM for protein domains (<https://pfam.xfam.org/family/> and MobiDB-lite for predicted disorder domains <http://old.protein.bio.unipd.it/mobidblite/>). Databases were accessed between the 15 – 20 January 2021. The Artemis comparison tool ACT was used for the genomic context of *secA1* and *secA2* genes.

**Supplementary Tables**

**Table S1. Determination of optimum fatty acid concentrations for resuscitation of DCTB in vitro.**

| Concentration ^$^  (µm) | Palmitoleic acid | Peteroselenic acid | Linolenic acid | Docosohexa-noic acid | Nervonic acid | Erucic acid | Eliadic acid |
| --- | --- | --- | --- | --- | --- | --- | --- |
| 0.125 | ND* | 3.30 ± 0.89 | 0.80 ± 0.75 | 5.27 ± 5.90 | 0.13 ± 0.23 | 8.93 ± 2.10 | 7.43 ± 0.51 |
| 0.25 | ND* | 3.23 ± 0.59 | 0.83 ± 0.76 | 5.37 ± 6.24 | 0.00 ± 0.00 | 8.77 ± 1.72 | 6.67 ± 2.26 |
| 0,5 | 1.10 ± 0.36 | 3.07 ± 1.03 | 0.80 ± 0.72 | 4.80 ± 5.48 | 0.07 ± 0.12 | 9.43 ± 2.50 | 7.93 ± 0.83 |
| 1 | 1.17 ± 0.29 | 3.60 ± 0.96 | 1.03 ± 0.47 | 5.17 ± 5.58 | 0.04 ± 0.06 | 9.93 ± 1.05^#^ | 7.53 ± 0.47 |
| 2,5 | 1.13 ± 0.21 | 4.47 ± 0.70 | 0.73 ± 0.64 | 5.20 ± 4.45 | 0.02 ± 0.03 | 9.40 ± 1.83 | 8.37 ± 1.76 |
| 5 | 1.13 ± 0.12 | 4.53 ± 1.46 | 1.43 ± 1.15 | 5.03 ± 4.48 | 0.00 ± 0.00 | 9.50 ± 2.29 | 8.30 ± 2.56 |
| 7,5 | 1.60 ± 0.17 | 4.10 ± 1.25 | 2.10 ± 1.18^#^ | 4.63 ± 4.43 | 0.00 ± 0.00 | 9.23 ± 1.63 | 7.07 ± 2.02 |
| 10 | 1.37 ± 0.15 | 3.23 ± 1.19 | 1.67 ± 1.08 | 4.40 ± 4.06 | 0.00 ± 0.01 | 9.47 ± 1.79 | 8.17 ± 1.91 |
| 12,5 | 1.40 ± 0.40 | 3.23 ± 1.11 | 2.07 ± 1.69 | 4.90 ± 4.06 | 0.00 ± 0.00 | 10.17 ± 2.22 | 8.27 ± 1.89 |
| 15 | 1.73 ± 0.38 | 4.23 ± 0.40 | 2.63 ± 2.83 | 7.23 ± 4.81 | 0.00 ± 0.00 | 11.30 ± 3.11 | 8.07 ± 1.53 |
| 17,5 | 1.83 ± 0.47 | 4.37 ± 0.71 | 2.30 ± 2.25 | 6.33 ± 6.70 | 0.14 ± 0.11 | 10.60 ± 3.73 | 8.07 ± 2.15 |
| 20 | 1.80 ± 0.44 | 3.77 ± 1.54 | 2.30 ± 2.00 | 4.60 ± 4.40 | 0.00 ± 0.00 | 9.97 ± 3.10 | 8.00 ± 2.63 |
| 22,5 | 1.73 ± 0.46 | 3.97 ± 0.76 | 2.57 ± 2.80 | 5.40 ± 5.82 | 1.07 ± 0.92 | 10.00 ± 2.60 | 7.87 ± 1.94 |
| 25 | 1.77 ± 0.42 | 4.10 ± 0.75 | 2.20 ± 2.84 | 4.97 ± 5.33 | 0.07 ± 0.06 | 10.33 ± 3.79 | 8.77 ± 2.47 |
| 27,5 | 2.07 ± 0.50 | 2.80 ± 2.42 | 2.33 ± 3.14 | 4.93 ± 4.93 | 0.00 ± 0.00 | 11.33 ± 3.79 | 7.27 ± 2.60 |
| 30 | 1.60 ± 0.44 | 4.07 ± 0.21 | 2.40 ± 3.41 | 5.50 ± 4.36 | 0.00 ± 0.00 | 10.80 ± 3.30 | 8.20 ± 0.44^#^ |
| 32,5 | 1.70 ± 0.35 | 2.57 ± 1.40 | ND* | 4.53 ± 3.97 | ND* | 11.37 ± 3.49 | 7.80 ± 1.82 |

^$^ Differentially culturable *Msm* were generated and resuscitated in 7H9 media supplemented with various concentrations of fatty acids. Resuscitation of the starved *Msm* cells was monitored by measuring the OD_600nm_ after 72 hrs. The optimal concentration for each of the fatty acids required to resuscitate DCTB in sputum was determined by calculating the average fold change in OD_600nm_ from three independent experiments with the standard deviation, as indicated by the shaded blue boxes.

# Whist this was not the highest bacterial recovery observed, this concentration was selected as it gave the least variation between biological repeats.

*ND – not done

**Table S2. List of qPCR primers used in this study**

| ***Gene*** | ***Primer Name*** | ***Primer Sequence (5’ – 3’)*** |
| --- | --- | --- |
| *sigA* | Msm_sigA-F  Msm_sigF-R | GGGCGTGATGTCCATCTCCT  GTATCCCGGTGCATGGTC |
| MSMEG_5700 (*rpfA^Msm^*) | Msm_rpfA-F  Msm_rpfA-R | CGTCATCTGCCAAGAACGTC  GGTGTTGATCGCCCAGTTG |
| MSMEG_5439 (*rpfB^Msm^*) | Msm_rpfB-F  Msm_rpfB-R | TCGCGCAATGCGAAGCGGGTGGTA  TGCCGGGATTGCGTCACCGTAGCA |
| MSMEG_4640 (*rpfE2^Msm^*) | Msm_rpfE2-F  Msm_rpfE2-R | TGGGGTGGTGGCGCGGGT  AGCAGGGTCGAGCACAGC |
| MSMEG_4643 (*rpfE1^Msm^*) | Msm_rpfE1-F  Msm_rpfE1-R | ACCTGGGCACCTGGCGGT  ACCGGCCAGGCGCCGATG |
|  |  |  |
| Rv0867c (*rpfA^Mtb^*) | Mtb_rpfA-F  Mtb_rpfA-R | CGGGTTATCGAACGCAACAC  GGTCGTTAGCGGCAAGTTCC |
| Rv1009 (*rpfB^Mtb^*) | Mtb_rpfB-F  Mtb_rpfB-R | TCGGATCAAGAAGGTCACCG  GCTACCGCGAACGTCACATC |
| Rv1884c (*rpfC^Mtb^*) | Mtb_rpfC-F  Mtb_rpfC-R | AGCTGCCTCTCGGGAACAAC  GACCACAGTGCGATCGGAAG |
| Rv2389c (*rpfD^Mtb^*) | Mtb_rpfD-F  Mtb_rpfD-R | GCAACAGATCGAGGTCGCAG  CGAGGAACGTCAGGATGTGG |
| Rv2450c (*rpfE^Mtb^*) | Mtb_rpfE-F  Mtb_rpfE-R | TGGCCTACAGCGTGAACTGG  GAAGCGAGCACGTTCTCAGC |

**Supplementary Figures**

**Figure S1: Domain organization of *Mtb* and *Msm* Rpfs.** Shown are the various domains found within the *Mtb* and *Msm* Rpf-like proteins, according to InterPro (<https://www.ebi.ac.uk/interpro/>). There is no direct homologue for *Mtb* RpfC and *Mtb* RpfD in *Msm*, instead *Msm* has a duplication of the *rpfE* gene and thus has two RpfE-like homologues. Green: signal sequence; Red: lytic transglycosylase domain (PF06737); Yellow: MobiDB-lite (disorder prediction); Blue: conserved domains DUF348 (PF03990) and G5 (PF07501). The domain structure was confirmed using <https://pfam.xfam.org/family/> and MobiDB-lite for predicted disorder domains <http://old.protein.bio.unipd.it/mobidblite/>. Signal peptides were confirmed at SignalP V5.0 (<http://www.cbs.dtu.dk/services/SignalP/>). *Msm* and *Mtb* Rpf sequences were obtained from <https://mycobrowser.epfl.ch/>.

**Figure S2: Identification of Sec-dependent signal peptides in *Mtb* and *Msm* Rpf-like proteins.** Signal sequence analysis was carried out using SignalP V5.0 (<http://www.cbs.dtu.dk/services/SignalP/>) which identified Sec dependent signal peptides (depicted by the black arrow) in *Mtb* RpfA, B, D and E, and all the *Msm* Rpf-like proteins. These proteins are predicted to be transported out of the cell.

*secA1* - *Rv3240c*


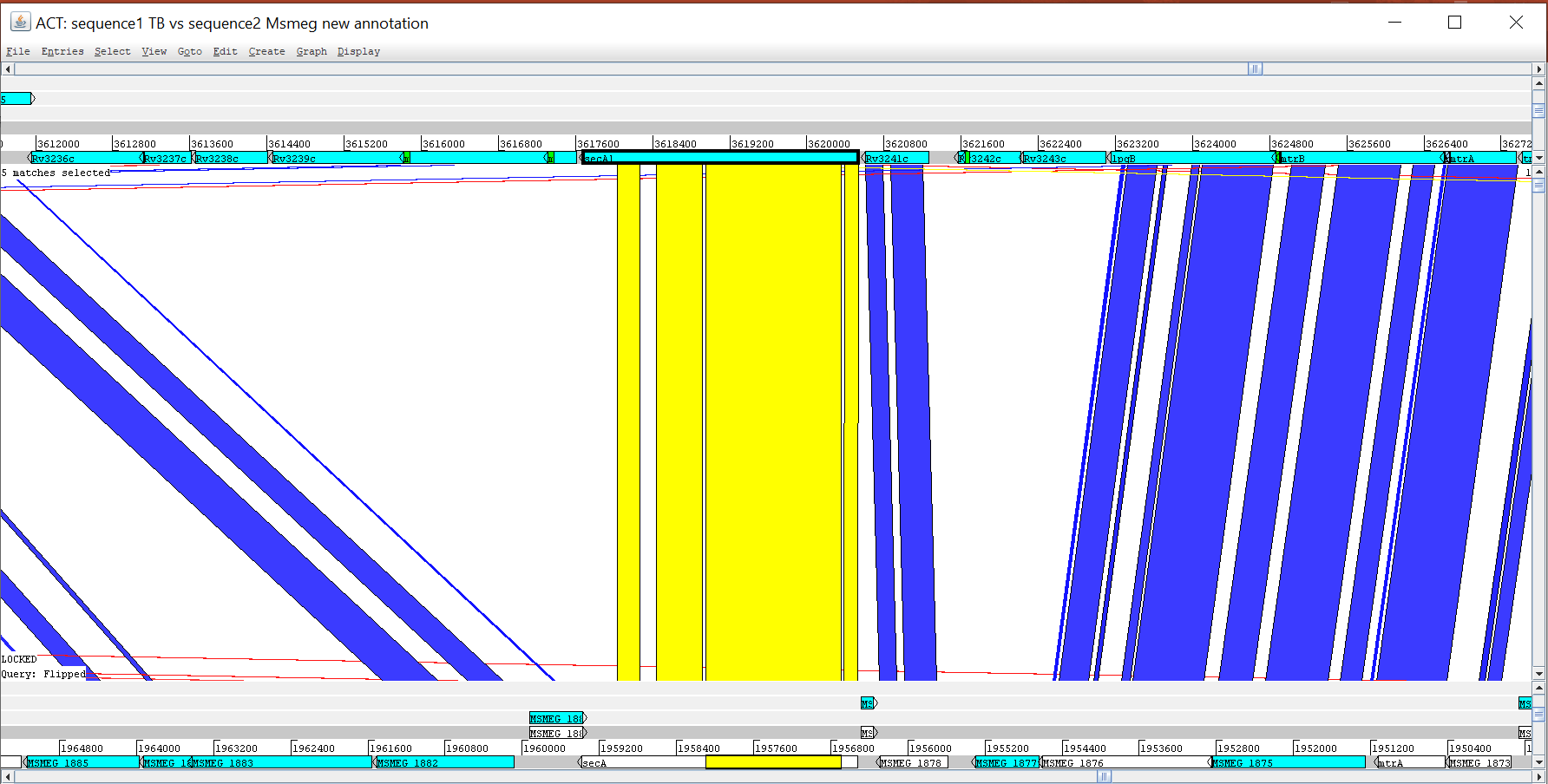


*Mtb*

*Msm*

*secA2* - *Rv1821*


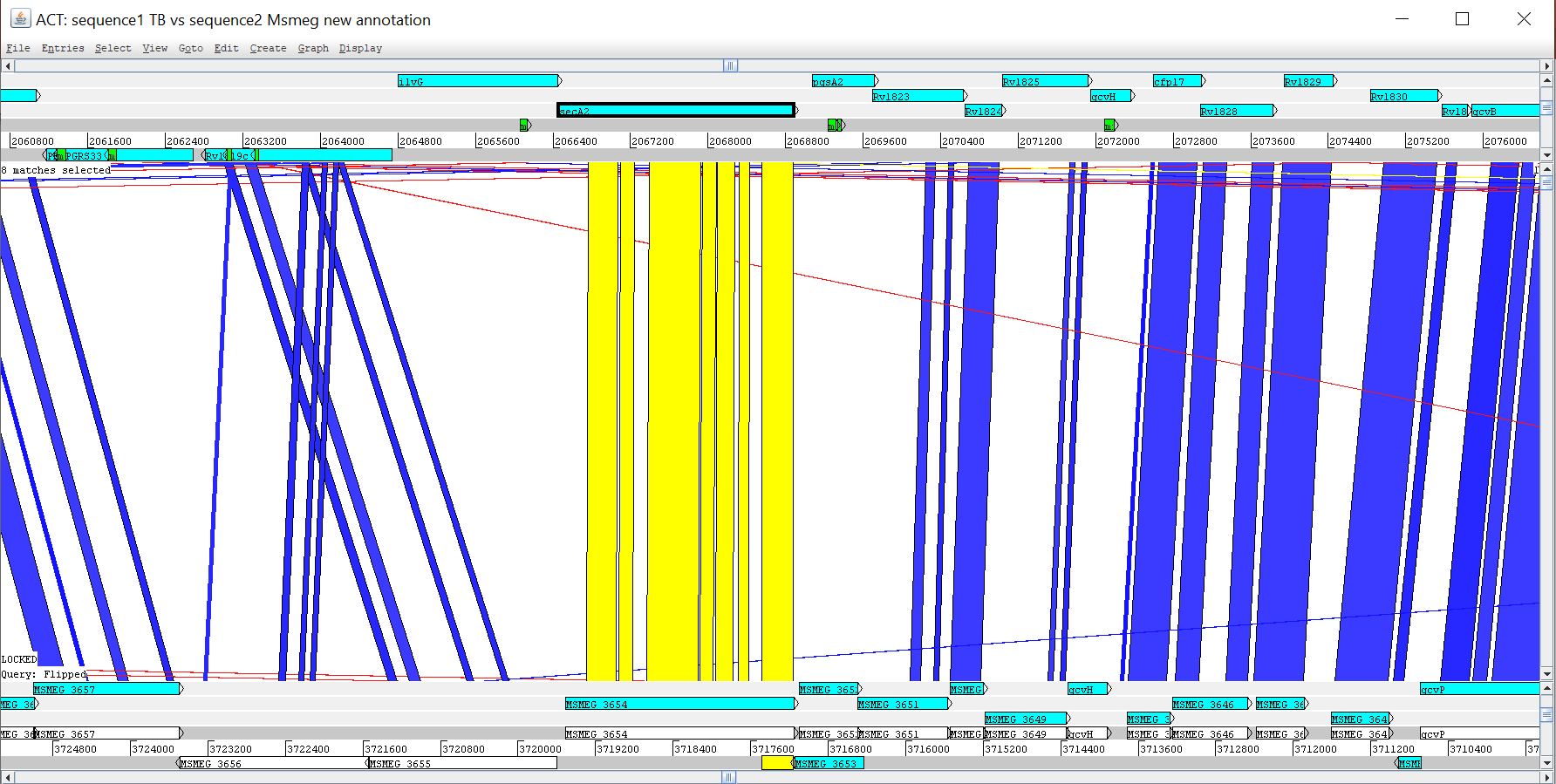


*Mtb*

*Msm*

**Figure S3: Comparative genetics of the *secA1* and *secA2* loci between *Mtb* and *Msm*.** The full genomes of *Msm* and *Mtb* were aligned using the Artemis comparison tool (ACT) followed by a detailed analysis of the *secA1* and *secA2* loci. Shown is the homology for these two loci between *Mtb* and *Msm*, both genes are conserved (shown in yellow). Scale – 16000 bp shown.

**References**

1 Kana, B. D. *et al.* The resuscitation-promoting factors of *Mycobacterium tuberculosis* are required for virulence and resuscitation from dormancy but are collectively dispensable for growth in vitro. *Mol Microbiol* **67**, 672-684, doi:MMI6078 [pii]10.1111/j.1365-2958.2007.06078.x (2008).

2 Ealand, C. *et al.* Resuscitation-Promoting Factors Are Required for *Mycobacterium smegmatis* Biofilm Formation. *Appl Environ Microbiol* **84**, doi:10.1128/AEM.00687-18 (2018).

3 Mukamolova, G. V., Turapov, O., Malkin, J., Woltmann, G. & Barer, M. R. Resuscitation-promoting factors reveal an occult population of tubercle Bacilli in Sputum. *Am J Respir Crit Care Med* **181**, 174-180, doi:200905-0661OC [pii]10.1164/rccm.200905-0661OC (2009).

4 Chengalroyen, M. D. *et al.* Detection and Quantification of Differentially Culturable Tubercle Bacteria in Sputum from Patients with Tuberculosis. *Am J Respir Crit Care Med* **194**, 1532-1540, doi:10.1164/rccm.201604-0769OC (2016).
